# Brain Trauma, Glucocorticoids and Neuroinflammation: Dangerous Liaisons for the Hippocampus

**DOI:** 10.3390/biomedicines10051139

**Published:** 2022-05-15

**Authors:** Ilia G. Komoltsev, Natalia V. Gulyaeva

**Affiliations:** 1 Department of Functional Biochemistry of the Nervous System, Institute of Higher Nervous Activity and Neurophysiology, Russian Academy of Sciences, 117465 Moscow, Russia; komoltsev.ilia@gmail.com; 2 Moscow Research and Clinical Center for Neuropsychiatry, 115419 Moscow, Russia

**Keywords:** hippocampus, brain trauma, glucocorticoids, corticosterone, cortisol, stress, neuroinflammation, neurodegeneration, remote damage

## Abstract

Glucocorticoid-dependent mechanisms of inflammation-mediated distant hippocampal damage are discussed with a focus on the consequences of traumatic brain injury. The effects of glucocorticoids on specific neuronal populations in the hippocampus depend on their concentration, duration of exposure and cell type. Previous stress and elevated level of glucocorticoids prior to pro-inflammatory impact, as well as long-term though moderate elevation of glucocorticoids, may inflate pro-inflammatory effects. Glucocorticoid-mediated long-lasting neuronal circuit changes in the hippocampus after brain trauma are involved in late post-traumatic pathology development, such as epilepsy, depression and cognitive impairment. Complex and diverse actions of the hypothalamic–pituitary–adrenal axis on neuroinflammation may be essential for late post-traumatic pathology. These mechanisms are applicable to remote hippocampal damage occurring after other types of focal brain damage (stroke, epilepsy) or central nervous system diseases without obvious focal injury. Thus, the liaisons of excessive glucocorticoids/dysfunctional hypothalamic–pituitary–adrenal axis with neuroinflammation, dangerous to the hippocampus, may be crucial to distant hippocampal damage in many brain diseases. Taking into account that the hippocampus controls both the cognitive functions and the emotional state, further research on potential links between glucocorticoid signaling and inflammatory processes in the brain and respective mechanisms is vital.

## 1. Introduction

Brain injury is a common cause of death and disability for people of all ages worldwide [1,2,3]. Depending on the biomechanics, brain lesions may occur both in areas of the brain directly adjacent to the place of force application and in remote areas [4]. The mechanisms of hippocampal damage are of particular importance, since they underlie late complications of traumatic brain injury (TBI), such as epilepsy, depression and cognitive impairment. The mechanisms of reorganization of neuronal networks in the hippocampus include long-lasting chronic neuroinflammation and secondary damage to the nervous tissue [5]. Responses and disturbances of the hypothalamic–pituitary–adrenal (HPA) axis may play a critical role in late post-traumatic pathology, in particular by modulation of synaptic activity and neuroinflammation in the hippocampus.

Even though stress-induced neuroinflammation and neurodegeneration in the hippocampus is fairly well described, and secondary hippocampal damage after TBI is studied by several groups, so far there have been no reviews on the glucocorticoid-dependent mechanisms of inflammation-mediated distant hippocampal damage with a focus on the consequences of TBI. PubMed search for the combination of words “TBI” (or “traumatic brain injury” or “brain trauma”) and “corticosterone” shows no review papers. A search query for “TBI” and “glucocorticoids” results in 15 relevant reviews, though none deals with the hippocampus. Ten of them are focused precisely on the clinical aspects of TBI: four reviews are related to pituitary disfunction after TBI [6,7,8,9]; three reviews are devoted to treatment [10,11,12], including usage of glucocorticoids (GCs) as medication; two reviews are focused on post-traumatic stress disorder [13,14]. Only five reviews include experimental studies; three of them are focused on the mechanisms of progesterone, dexanabinol and dexamethasone effects [15], TNF inhibitors [15] and mesenchymal stem cells in TBI [16]. One review describes the blood–brain barrier disfunction and the effects of GCs on its permeability [16]. The last and most relevant review was published in 2019 and was devoted to pro- and anti-inflammatory action of GCs after TBI [17]. Nevertheless, it lacks several important aspects of GC action on synaptic transmission and excitotoxicity in the hippocampus.

The aim of the present review was an analysis of the glucocorticoid-dependent mechanisms of inflammation-mediated distant hippocampal damage with a focus on the consequences of traumatic brain injury. In addition, we have systematically analyzed relevant papers resulting from a PubMed search for the combination of “TBI” and “corticosterone”.

## 2. TBI, Its Late Consequences and the Hippocampus

Post-traumatic epilepsy (PTE) is a severe complication of traumatic brain injury (TBI). It occurs in 10–20% of patients after TBI [18]. About 57% of patients with PTE suffer from medial temporal lobe epilepsy diagnosed by the semiology of epileptic seizures, signs on the EEG and MRI [19]. Histological analysis reveals a specific epilepsy-related pathology of the hippocampus, hippocampal sclerosis, in at least half of these patients [20]. The main risk factors for PTE include the severity of injury, subdural, intracerebral hematomas and early seizures [18,21,22,23,24].

Major depressive disorder development after TBI is quite frequent. Over 50% of patients met major depression disorder criteria at least once, the disease being associated with poorer health-related quality of life [25]. Pathological changes in the hippocampus may represent a basis for post-traumatic depression [26], and, taking into account the common pathophysiological mechanisms, depression is considered the main comorbid pathology for epilepsy [27]. Interestingly, anxiety and depression in patients can be diagnosed before the diagnosis of epilepsy [28], thus confirming common bases of disease mechanisms but not a unidirectional causal relationship between depression and epilepsy.

The mechanisms of late TBI complications and reorganization of neuronal networks in the hippocampus include long-lasting chronic neuroinflammation and secondary damage to the nervous tissue [5]. The causes of chronic neuroinflammation development and circuit reorganization are obviously complicated and, so far, remain obscure. Recent data suggest that disturbance in the HPA axis function plays a critical role in late post-traumatic pathology.

## 3. HPA Axis in Patients with TBI

**Figure 1:**
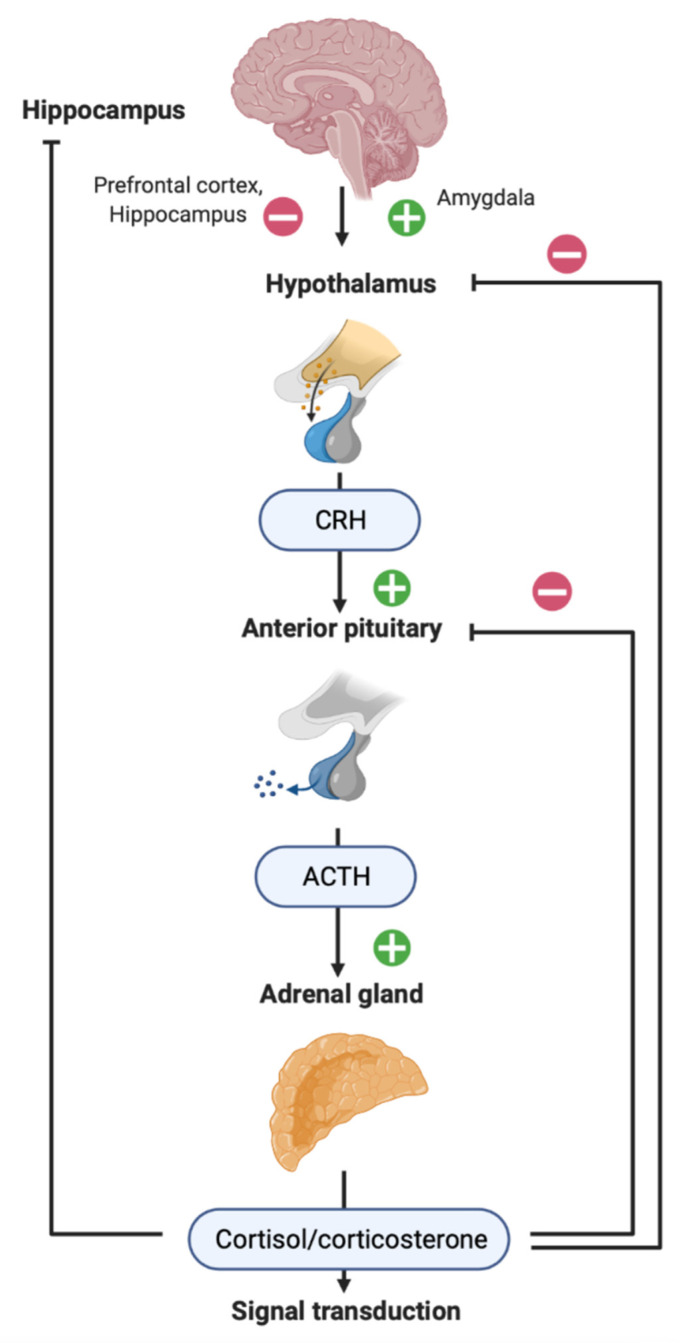
Hypothalamic–pituitary–adrenal (HPA) axis. Neuroendocrine response to stress includes the reaction of HPA axis: the release of hypothalamic corticotropin-releasing hormone (CRH), which stimulates the release of adrenocorticotropic hormone (ACTH) from the pituitary gland and, finally, the release of glucocorticoids (GCs) from the adrenal glands (corticosterone in most rodents; cortisol in humans). GCs enter the blood circulation, implementing both peripheral and central action via specific receptors in almost all organs and tissues, including the brain. The prefrontal cortex, hippocampus and amygdala control the activity of the hypothalamus, thus regulating the HPA axis [29,30,31].

HPA axis (Figure 1) is the main neuroendocrine system of the organism implementing stress response and controlling adaptive mechanisms at different levels, from subcellular to the whole organism [29,30,31]. Normally, physiological stress is realized due to HPA action and release of glucocorticoids (GCs). Clinical studies of cortisol-dependent damage in TBI are limited, and the data are scarce and contradictory. Different groups report that cortisol level after TBI is decreased [32] or increased [33,34]. TBI is an acute physiological stress and is expected to increase cortisol levels, at least in TBI patients with preserved HPA axis function. However, some patients with TBI develop dysfunction of the anterior or posterior pituitary gland, which, in turn, leads to secondary hypocorticism (a decrease in cortisol levels due to a decrease in the production of pituitary adrenocorticotropic hormone, ACTH). Agha et al. [35] showed that ACTH and cortisol production after stimulation by glucagon in patients with TBI may be normal or reduced. In patients with a reduced response, the basal cortisol level after TBI was also decreased, but in patients with a normal response, it was increased. The risk factors for adrenal insufficiency and a decreased cortisol level in the acute period of TBI are basal skull fractures, hypotension and the use of propofol [36]. Hydrocortisone replacement therapy may be associated with a favorable neurologic outcome after TBI, suggesting the involvement of corticosteroids in the consequences of brain trauma [37]. In general, signs of mild TBI, including absence of amnesia and a higher Glasgow coma scale score, are associated with higher cortisol levels [38], while the severity of coma positively correlates with acute cortisol level (within 6 h after TBI) [34]. On the contrary, during the first 3 days after TBI, the cortisol level is higher in patients with lower Glasgow coma scale score and predicts mortality [39].

The time course of cortisol level during the first weeks after TBI also depends on the initial HPA axis state. In patients with stressful events prior to brain injury, cortisol levels were significantly decreased, as compared with patients without stress before TBI [40], indicating stress-induced HPA dysfunction. In patients without stressful events before TBI, HPA function was preserved, and GC levels increased. Chronic HPA disturbances in patients with TBI are studied even less. In mild TBI, hair cortisol did not diverge before and months after TBI, its level reflecting individual coping with stress in general [41]. However, HPA dysregulation was shown two years after TBI, when hypocortisolemia and low diurnal GCs variability were detected [42]. Another study reported normal cortisol and circadian variations for two years after mild-to-moderate TBI, even with the presence of depression [43].

According to the data of basic experimental research, it can be assumed that an altered physiological response to acute stress may underlie some long-term effects of TBI. However, convincing clinical studies in this area are still lacking. Corticoid-related primary and secondary mechanisms of TBI, studied in animal models, are discussed below.

## 4. Distant Hippocampal Damage in Rodent TBI Models

Lateral fluid percussion brain injury model in rats [44,45] or mice [46,47] is the most conventional TBI model. A “golden standard” of TBA modeling, it allows studying the mechanisms of primary and secondary brain damage induced by TBI, though a few other models are also used [48].

Primary damage includes direct impact to brain tissue, which is accompanied by rupture of cell membranes, mechanical disruption of the blood–brain barrier, release of albumin and other blood components into the extracellular space. Acute damage causes severe metabolic disturbances inducing deficits in ATP production, energy deficiency and subsequent impairment of Na^+^/K^+^ ATPase, as well as an increase in the concentration of extracellular K^+^. Changes in the extracellular K^+^ cause depolarization of neuronal membranes and additional opening of voltage-gated calcium channels (VGCC), neurotransmitter release and a fast increase in the level of excitatory amino acids in the extracellular space [49]. Continuous changes in the concentration of extracellular ions further reduce the threshold of neuronal excitability and are aggravated by their repeated excitation. In addition, energy deficiency leads to the generation of free radicals and reactive oxygen species involved in oxidative stress and secondary damage to the nervous tissue [49]. Primary damage results in continued metabolic changes, excitotoxicity and the edema formation, inflammation, apoptosis and necrosis, representing the mechanisms of secondary brain damage.

TBI applied to the neocortex induces secondary, distant damage to the hippocampus. Neuronal death and glial activation are detected in the CA3 field and the dentate gyrus (DG) of the hippocampus [50]. Less pronounced changes are detected in the contralateral hippocampus [51,52]. Bilateral changes in the hippocampus after repeated TBI were also shown [53]. GABAergic neurons (parvalbumin (PV), calretinin, somatostatin and neuropeptide Y-immunoreactive) in the polymorphic layer of the DG are among the most vulnerable populations of hippocampal neuronal cells. Previously, we described the development of distant hippocampal damage after lateral fluid percussion brain injury in rats [54,55]. Selective neuronal cell loss in the polymorph layer of the hippocampal DG was demonstrated bilaterally; in the ipsilateral hippocampus, it was evident on day 3, but in the contralateral hippocampus, these changes were delayed and detected on day 7. Microglial activation was evident in the hippocampus bilaterally on day 7 after TBI, while pro-inflammatory cytokines mRNA levels increased bilaterally from day 1 after TBI.

It is worth noting that distant damage to the hippocampus has been reported to be a result of different extremal factors, including brain ischemia [31]. Remote hippocampal damage is a well-documented consequence of chemoconvulsant injection (kainate [56], dendrotoxin [57], pentylentetrazole [58,59]). Neuronal death [60], excitotoxicity and the involvement of glutamate receptors in distant hippocampal damage were shown after TBI in rats [61,62], indicating the similarity of damage mechanisms, irrespective of primary injury nature. In general, many epileptogenic lesions are characterized by secondary neuronal death in the DG, both in experimental and clinical settings [63]. The involvement of both NMDA and non-NMDA receptors [53,56], excitotoxicity and spreading of epileptiform activity are [64,65] discussed as mechanisms of the damage. Acute and excessive release of glutamate and aspartate leads to the activation of glutamate receptors (primarily AMPA receptors) and depolarization of neuronal membrane. Activation of glutamate, kainate and calcium-permeable AMPA receptors contributes to an increase in intracellular levels of calcium, a universal secondary messenger [49]. Excessive intracellular calcium concentration activates phospholipases, endonucleases and proteases (calpains), accelerating neuronal death as a result of excitotoxicity by apoptotic and necrotic mechanisms [66,67,68].

Thus, excessive glutamate level after TBI is a trigger for secondary neurodegeneration, which involves death of GABAergic neurons. Based on (1) the relative selectivity and distant character of hippocampal damage; (2) lack of specificity regarding the type of primary impact inducing distant hippocampal damage and (3) the involvement of both ipsilateral and contralateral hippocampus, it can be assumed that there are common ***systemic*** mechanisms underlying selective death of hippocampal neurons and, possibly, the development of chronic neuroinflammation. This selectivity may be explained by the effects of GCs, affecting the hippocampus and functional properties of the hippocampal networks [26,31].

## 5. Glucocorticoid Signaling, Hippocampus and Neuronal Death

GCs (corticosterone, CS, in most animals, and cortisol in humans) are essential hormones in all vertebrates, regulating two basic systems: glucose metabolism and immune response. In general, GCs suppress inflammation and increase blood glucose level by stimulating gluconeogenesis and inhibition of glucose uptake by cells [69]. Specific receptors mediating signals of GCs are present in most cells of the organism. GCs regulate the behavioral response to stress, and their receptors are widely expressed in the brain. The effects of GCs are critically determined by the specific aspects of their action [70]. During the stress response, GCs modulate the hippocampal function, affecting numerous signaling and metabolic systems [31]. It is also important that, unlike other brain structures, the basal membrane covers only 30% of the vascular surface in the hippocampus [71], which facilitates the penetration of hormones into hippocampal neurons.

**Figure 2:**
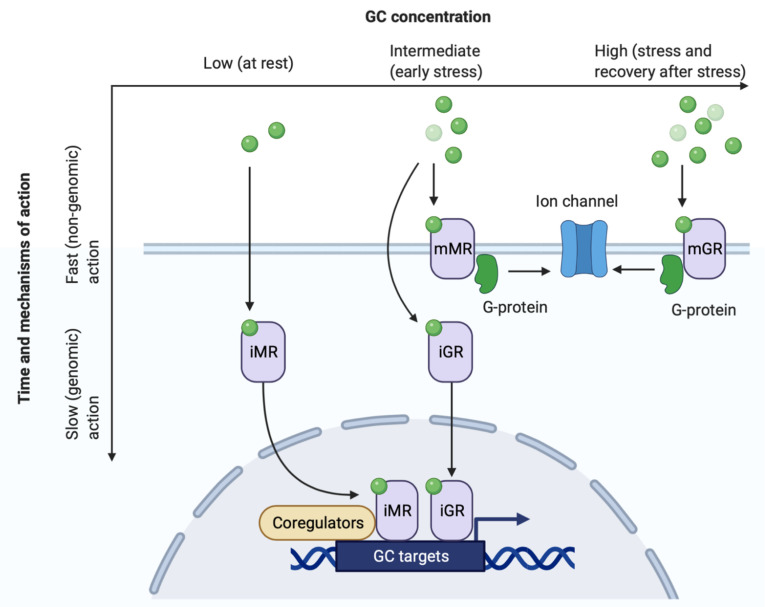
Receptors of glucocorticoids [29,30,31,83]. See details in Chapter 5. Green circles—glucocorticoid molecules.

The functions of the GCs in the hippocampus are mediated by high-affinity mineralocorticoid (MR) and low-affinity glucocorticoid receptors (GR). MRs bind GCs at low hormone levels, while the affinity of GRs to GCs is much lower, and the activation of these receptors occurs when GC levels increase, for example, during stress response. Each type of corticosteroid receptor is presented by two forms: intracellular cytoplasmic/nuclear receptors, exerting primarily slow genomic action (iMR, iGR), and non-genomic membrane-bound receptors (mMR, mGR), rapidly altering excitatory neurotransmission [29,72,73]. The affinity of membrane-bound and intracellular receptors decreases in the order: iMR > iGR = mMR > mGR [30] (Figure 2). The genomic binding loci of GR and MR comprise hundreds of partially overlapping DNA sites changing during the circadian cycle and stress [74]. Intracellular MRs and GRs translocate to the nucleus, where they act as nuclear transcription factors and modify gene expression, affecting protein synthesis. The genomic effects are realized within hours and may persist for many days, underlying adaptation, synaptic and cellular plasticity. Membrane-associated GRs and MRs act through G-proteins and affect the ion channels, rapidly modulating cell excitability. In general, the effects of GCs on specific cell populations depend on: (1) their concentration, (2) duration of exposure, (3) cell type with definite balance of specific intracellular and extracellular GRs and MRs.

MRs are expressed mainly in the brain regions that are crucial to the formation of memory and emotions, such as the hippocampus, amygdala, frontal, enthorinal and insular cortex. GCs trigger rapid non-genomic effects on excitability of neurons in brain through mMRs, thus influencing the cognitive and emotional functions and adaptive behavior within minutes. Besides limbic structures, GRs are also expressed in the prefrontal cortex and are involved in cognitive and executive functions, such as reasoning and attention [30,75]. GRs are involved in negative feedback on HPA axis; the amygdala stimulates the HPA axis, whereas both the hippocampus and prefrontal cortex have inhibitory effects [76,77]. It is noteworthy that after TBI in rats, CRH may increase the excitability of the amygdala and hippocampus [78].

The group of R. Sapolsky studied the effects of stress in animals for many years and showed that sustained exposure to stress induces neuronal loss in the hippocampus [79,80]. Chronic stress resulted in about 20% loss of neurons in CA3 field of the hippocampus of rats, and GCs also worsened other types of damage produced by ischemia, seizures or excitatory amino acids in CA1 and CA3 fields. The authors explained these effects by suppression of glucose transport, changes in calcium metabolism and suppression of neurotrophic factors expression [79,81,82]. Since neuronal energy metabolism is almost exclusively dependent on oxidative phosphorylation, and neurons have almost no glucose storage, they are the cells most vulnerable to energy restriction. Adaptive processes are highly energy dependent, and GCs may worsen neuronal survival.

Another mechanism of GCs-dependent neurodegeneration may be the elevation of intracellular calcium and glutamate excitotoxicity. GCs dose-dependently affect the excitability of neurons: glutamatergic synaptic transmission is enhanced by intermediate doses of GCs acting on mMR (rapid response to stress) and is reduced by higher doses acting on GR in addition to the already activated MRs (recovery after a stressful situation) [72,84] (Figure 2).

**Figure 3:**
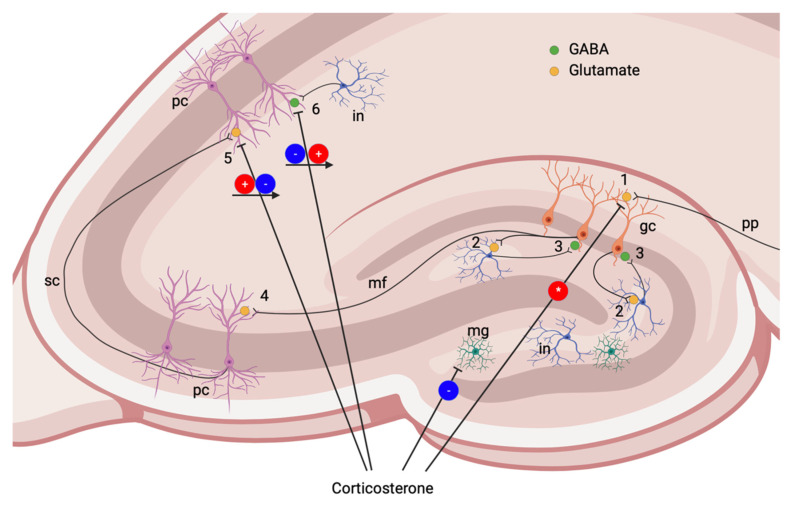
Corticosterone effects in the hippocampus at rest and during acute stress. 1, Glutamatergic synapse on granular cell. In normal conditions, granular cells are almost insensitive to physiological GSs changes, including acute stress, but extremely low levels of GCs reduce neuronal activity of granular cells [88]. 2, Glutamatergic collateral to an interneuron. 3, GABAergic synapse on granular neuron. Though little data on the modulation by GCs of collateral inhibition in the DG are available, the effects of GCs on pyramidal cells of CA1 field may be similar. 4, Glutamatergic synapse on pyramidal neuron of CA3 field. 5, Glutamatergic synapse on pyramidal neuron of CA1 field. Pyramidal neurons demonstrate U-shaped modulation by GCs: very low (not physiological), as well as very high CS levels suppress neuronal activity, but intermediate doses increase EPSP amplitude [83,85,86]. 6, GABAergic synapse on pyramidal neuron. GCs may temporarily reduce IPSP with subsequent rapid or slow elevation of inhibitory postsynaptic potential (IPSP) amplitude [93,94]. pp, perforant path; gc, granular cell; mf, mossy fibers; pc, pyramidal cells (CA3 and CA1 fields), sc, Schaffer collateral; in, interneuron. Red circle (+)—activating action; red circle (*)—activation by very low GC levels; blue circle (−)—inhibiting action; arrows show changes in GC action with increasing concentration or over time.

**Figure 4:**
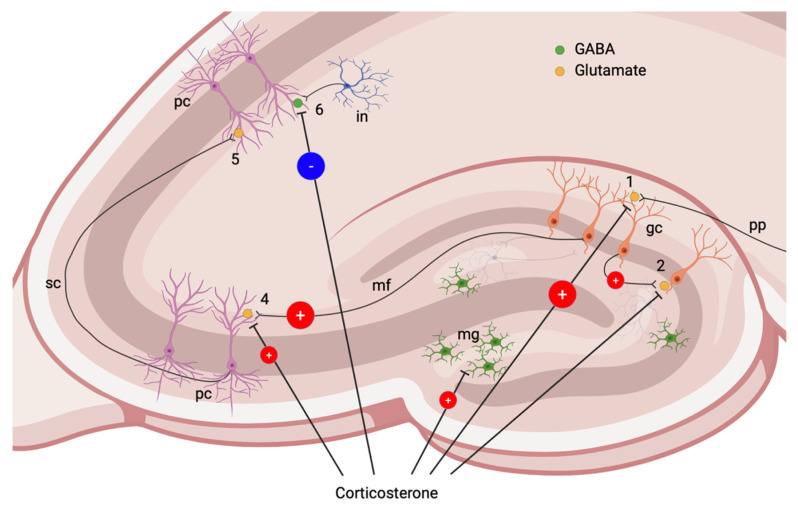
Effects of GCs in chronic stress and structural post-traumatic changes in the hippocampus. GABAergic neuronal loss in the DG and neuroinflammation are histological hallmarks of late post-traumatic changes. 1, Glutamatergic synapse on granular cell. GCs enhance glutamatergic AMPA-mediated signaling [89]. 2, Glutamatergic collateral on granular neuron (mossy fiber sprouting) enhances DG excitability [97]. 4, Glutamatergic synapse on pyramidal neuron of CA3 field. Chronic stress increases EPSP amplitude via NMDA-dependent signaling [87]. Failure of inhibition due to GABAergic neuronal loss is demonstrated [50,98]. 5, Glutamatergic synapse on pyramidal neuron of CA1 field. Though little data on the modulation by chronically elevated GCs of glutamatergic synapses in the CA1 are available, the effects of GCs on pyramidal cells of CA3 field may be similar. 6, GABAergic synapse on pyramidal neuron. Rhythmic IPSCs due to loss of interneurons are demonstrated [94]. pp, perforant path; gc, granular cell; mf, mossy fibers; pc, pyramidal cells (CA3 and CA1 fields); sc, Schaffer collateral; in, interneuron. Red circle (+)—activating action; blue circle (−)—inhibiting action.

The influence of GCs on neuronal excitability was extensively studied by the M. Jöels group. The effects of GCs on CA1 pyramidal neurons are explained by a U-shaped curve: very low (not physiological, e.g., after adrenalectomy), as well as very high CS levels suppress neuronal activity, but intermediate doses increase the amplitude of excitatory postsynaptic potentials (EPSPs) [83,85,86] (Figure 3). In animals subjected to chronic stress, changes of pyramidal neurons are rarely found. In pyramidal neurons of CA3 field, EPSP amplitude increased, likely due to NMDA action in chronically stressed animals [87] (Figure 4).

In granular neurons of the DG, MR-dependent effects on field potentials caused by the activation of AMPA receptors were demonstrated [83,85]; however, they are almost insensitive to physiological GS changes, including acute stress. In adrenalectomized rats, extremely low level of GCs reduced neuronal activity of granular cells [88] (Figure 3). In contrast, in animals exposed to chronic stress, GCs enhanced glutamatergic AMPA-mediated signaling in granular cells [89] (Figure 4).

Voltage-gated calcium channels (VGCC) are among the principal players involved in the control of calcium homeostasis. They are activated at depolarized membrane potentials and become permeable for calcium. Amplitudes of VGCC currents increased after 1–4 h of exposure to CSs, more likely through iGR signaling [90]. In chronic stress, GCs also increased calcium currents through VGCC, both in granular cells of the DG [91] and in pyramidal cells of CA1 field [92]. Thus, VGCC activation increases risk of cell death at glutamate excess, especially in chronic stress. 

Thus, GC excess plays an essential role in the selective vulnerability of the hippocampus, promoting calcium overload, energy deficits and secondary death of neurons [95] by increasing the susceptibility of neurons to glutamate excitotoxicity [96]. Importantly, glutamatergic axons terminate on interneurons (Figure 3), and excitotoxic damage affects primarily GABAergic interneurons, the most vulnerable population of cells in the hippocampus.

On the other hand, GCs impair the protective activation of inhibitory neurotransmitter systems during insults [79]. Patch-clamp recordings show that GCs, through the activation of MRs, reduced the frequency of spontaneous inhibitory postsynaptic potentials (IPSP) in pyramidal cells of CA1 field of the ventral hippocampus (more likely due to membrane-associated receptors) but not in the dorsal part. The effect of a GR agonist was different: it slowly increased IPSP magnitude in the hippocampus, more likely through iGRs [93]. Another group demonstrated rapid increase in spontaneous inhibitory postsynaptic currents (IPSCs) via mGR in CA1 pyramidal cells [94] (Figure 3). The authors explained that the controversial result of rapid GC action is likely due to the difference in experimental conditions. 

In chronic stress, rhythmic IPSCs originating from the PV-positive GABAergic neurons was impaired due to selective PV-positive cell loss, demonstrating lack of inhibition in CA1 [94] (Figure 4). The authors explained the selective loss of PV-positive neurons by the sustained activation of interneurons and imbalance in perisomatic inhibition.

Thus, GCs modulate the excitability of the hippocampus in acute and chronic stress and enhance glutamate excitotoxicity, potentially causing selective neurodegeneration in the hippocampus. Since TBI increases GC levels in humans and in animal models [54,55], the consequences of stress can be considered as one of the secondary brain injury mechanisms.

## 6. Neuroinflammation and TBI

Both primary and secondary mechanisms of brain damage discussed above are associated with neuroinflammatory response. Neuroinflammation is one of the essential mechanisms of brain damage modulated by GCs. Under normal conditions, microglial activity and cytokine release are intimately associated with neuroplasticity and memory; however, uncontrolled excessive neuroinflammation leads to cell death and further progression of brain pathology [99]. In pathological conditions, microglia play a crucial role in the expression of both pro-inflammatory (IL-1ß, IL-6, TNFα) and anti-inflammatory (IL-4, IL-10) cytokines, chemokines, as well as molecular fragments associated with damage (DAMP, damage-associated molecular patterns, such as HMGB1, ATP, S100ß). These substances enable microglial modulation of cyclooxygenase-2 and the components of the complement system [49]. Cytokines produced by inflammatory cells are released within minutes after TBI and alter the functioning of glutamate and GABAergic receptors, as well as potential-dependent ion channels, inhibit the reuptake of glutamate by astrocytes and provoke an increase in extracellular K^+^. Thus, cytokines can participate in hypersynchronization of neurons and the occurrence of epileptiform activity [100], as well as contribute to further neurodegeneration [101]. Microglial activation also occurs in remote areas of the brain, microglial properties and cytokine profile changing over time [102].

In the area of TBI, neutrophils and other cells of the immune system are recruited as well. These cells, along with glia, take part in the production of cytokines, chemokines, free radicals, prostaglandins and components of the complement system. The profile of peripheral immune system cells changes over time. Neutrophils first appear in the focus of injury; after 3–5 days they are replaced by mononuclear leukocytes and, to a lesser degree, by T cells, dendritic cells and natural killers [49]. The peripheral immune system is also activated. It has been shown that 1 day after TBI, the number of CD4+ and CD8+ T cells in rat spleen increases, indicating an activation of adaptive immunity. Suppression of adaptive immunity improves TBI outcomes [103]. Thus, autoimmune mechanisms are involved in the development of post-traumatic pathology [49], though their role has not been studied in detail yet.

Astrocytic gliosis in the neocortex develops about 1 week after TBI and, in the long-term period of injury astrogliosis, serves as an important histopathological marker of hippocampal sclerosis [104,105,106]. Astrocyte dysfunction may be involved in increasing the excitability of neurons and circuit reorganization via several mechanisms. Astrocytes normally participate in the utilization of extracellular K^+^ (due to active transport into the cell and distribution through the astrocyte system) and utilization/metabolism of glutamate. Changes in K^+^ homeostasis and an increase in its concentration lead to a decrease in neuronal excitability threshold, while impairment of glutamate utilization results in an increase in its toxic effects. In addition, astrocytes play an important role in water homeostasis of the brain [107] and form the brain glymphatic system involved in the development and resorption of edema, transport of metabolites and immune cells [108].

The transition from acute activation of the brain immune system to chronic neuroinflammation in TBI is the subject of quite a few studies [5,17,109]. Chronic neuroinflammation caused by TBI induces progressive edema and neurodegeneration associated with cognitive and emotional disorders [110]. The first week after TBI is an important time interval, day 7 being considered a borderline between acute and chronic post-traumatic changes. It is noteworthy that edema resorption and the early development of astrogliosis in the focus of direct impact to the neocortex was shown 7 days after TBI [104,105,106]. 

## 7. Neuroinflammation and GCs

Chronic neuroinflammation is a recognized consequence of chronic stress; its definitive association with GCs is rigorously discussed but still remains obscure [111]. The available data indicate dual effects of GCs, both anti- and pro-inflammatory. Suppression of inflammation is among the well-established systemic effects of GCs. This ability of GCs is widely used in clinical practice for treatment of inflammatory and autoimmune diseases. The activation of GRs and MRs in peripheral tissues results in inhibition of immune cell activity and induction of apoptosis in lymphocytes [112]. GCs also inhibit inflammation via several other mechanisms, including inhibition of tissue infiltration by cells from the blood, inhibition of cytokine expression, changes of lymphocyte functioning and others [111]. In the brain, GCs realize either pro- or anti-inflammatory properties depending on the degree and duration of exposure, external factors preceding injury, injury characteristics and the specific brain region [31,70,111]. 

**Figure 5:**
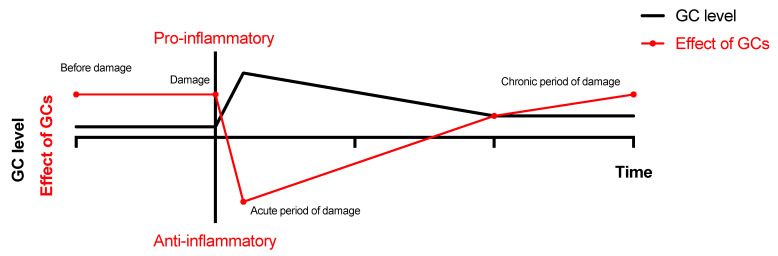
Effects of GCs on neuroinflammation depend on time of damage [54,55,111,113]. Timing of GCs exposure is critical for its pro- or anti-inflammatory action in the brain.

The order and time period between GC increase and immune challenge may be important for the effects of GCs on neuroinflammation (Figure 5). This was confirmed in a study with administration of GCs and lipopolysaccharide (LPS, immunogenic component of Gram-negative bacteria) in a different order [113]. If GCs were injected prior to LPS (2 and 24 h), they potentiated pro-neuroinflammatory effects (TNFa, IL-1b, IL-6 expression). In contrast, GCs injected 1 h after LPS had an anti-inflammatory action in the brain. LPS injection directly into the hippocampus of the stressed animals also increased the number of reactive microglial cells and expression of pro-inflammatory cytokines [114] as compared to non-stressed animals. The second factor affecting GCs action is the duration of their exposure (Figure 5). Many groups have demonstrated that chronic stress is definitely a pro-inflammatory condition [111]. Chronic stress potentiated LPS-induced activation of several pro-inflammatory pathways, including nuclear factor kappa B (NF-κB) [115], and increased basal activation of other intracellular pathways, such as ERK1/2, p38, SAPK/JNK and AKT [116]. 

The interaction between GCs and the inflammatory mechanisms seems really intricate. Dexamethasone injected directly into the rat hippocampus was able to induce weak neuroinflammation but, when applied during LPS-induced neuroinflammation, evoked differential effects on pro-inflammatory cytokines expression [117]. Systemic administration of dexamethasone for 3 weeks in mice, mimicking chronic stress, induced depressive-like behavior and glucocorticoid resistance, a potential priming factor enhancing inflammatory response [118]. After ten days of corticosterone exposure in adrenalectomized rats, GCs, in a dose-dependent manner, primed microglia to pro-inflammatory stimuli by gene expression associated with inflammation (NLRP3, Iba-1, MHCII and NF-κB), thus potentiating microglial pro-inflammatory response to LPS [119]. Interestingly, diffuse TBI also primes microglia and promotes depressive-like behavior after secondary LPS-induced inflammatory challenge 1 month after trauma [120].

Recent information about relationships between inflammation, GCs and TBI is scarce. CS increased 1 hour after TBI, and its level negatively correlated with the number of peripheral T cells, confirming the anti-inflammatory effect of GCs [121]. The number of circulating T cells positively correlated with TBI core infiltration and destructive neuroinflammatory response in the brain.

**Figure 6:**
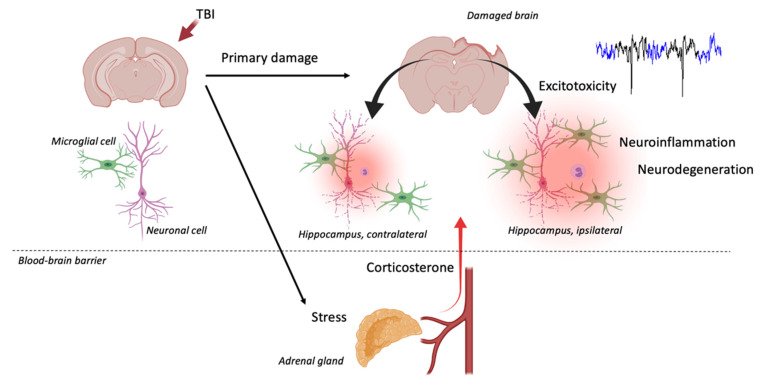
Local and systemic effects of TBI ([54,55]). Based on (1) selectivity and distant character of hippocampal damage, (2) lack of specificity to the type of primary impact leading to distant hippocampal damage and (3) involvement of both ipsilateral and contralateral hippocampus in models of unilateral primary neocortical injury, it can be assumed that there are common CS-dependent mechanisms underlying selective death of hippocampal neurons and chronic neuroinflammation.

Using the lateral fluid percussion model of TBI, we showed CS elevation in the blood and the hippocampus on day 3 after TBI [54,55]. The correlations between CS and neuroinflammatory response in the hippocampus were time dependent and vague. On day 3, the blood CS level negatively correlated with microglial cell count in the hippocampus. In contrast, on day 7 after TBI, when CS almost returned to baseline, noticeable and bilateral microglial activation was detected. The levels of IL-1β in the contralateral hippocampus positively correlated with CS in the same region. These results may reflect an early anti-inflammatory and latter pro-inflammatory effect of CS in TBI (Figure 6).

Since GCs modulate the secondary mechanisms of damage, the HPA axis state during trauma is also an important factor for GC action. On the one hand, the time course of cortisol levels after TBI depends on the initial HPA state; in patients experiencing stressful events before brain injury, acute cortisol levels significantly decreased during the acute period of TBI [40]. Thus, patients with previously activated HPA demonstrate impaired stress reactivity. This may defeat the positive effects of GCs (early anti-inflammatory action) and enhance negative ones (e.g., enhancement of excitotoxicity). On the other hand, moderate stress may increase the resistance of neurons to brain insults and protect from further excitotoxic damage; the expression of cytokines and neurotrophic factors may underlie the protective effects of mild stress [122].

## 8. CS Changes and Associated Events in Animal Models of TBI: Summary Table

Additionally, we have summarized the data generated from systematic analysis of all 48 relevant papers resulting from the PubMed search for the combination of “TBI” and “corticosterone” (21 of them combined with “hippocampus” and 7 of them with “neuroinflammation”). The results on CS changes are presented in Table S1 (Supplementary Material). In addition to CS alterations, the cellular, molecular and behavioral changes revealed in these papers are shown in the last column. The data are different and sometimes appear contradictory; however, the main reasons for discrepancies seem to be significant differences in the aims of the studies and, hence, in the experimental designs used by different groups.

## 9. Conclusions: TBI and Beyond

In this review, we discussed GC-dependent common mechanisms of stress- and inflammation-mediated distant hippocampal damage, focusing on the consequences of TBI. The effects of GCs on specific neuronal populations in the hippocampus depend on GC levels, duration of GC exposure and cell type (in particular, the balance of specific intracellular and extracellular GR and MR). Pro- or anti-inflammatory effects of GCs also depend on their concentration and exposure duration. Previous stress and elevated GC level prior to pro-inflammatory impact may inflate pro-inflammatory effects. Long-term and moderate elevation of GCs may also enhance neuroinflammatory response. GC-mediated long-lasting neuronal circuit changes in the hippocampus after TBI are involved in late post-traumatic pathology development, such as epilepsy, depression and cognitive impairment. Complex and diverse actions of HPA axis on neuroinflammation may be essential for late post-traumatic pathology.

Importantly, these mechanisms are applicable to remote hippocampal damage occurring after other types of focal brain damage (stroke, epilepsy) or central nervous system diseases without obvious focal injury (e.g., infections). Secondary damage to the hippocampus is shown in the middle cerebral artery (MCAO) model in rats [123,124]. MCAO induces accumulation of the pro-inflammatory cytokine IL-1β accompanied by elevated CS at the early and delayed stages of stroke [124]. High initial level of GCs and previous stress exacerbate damage to the hippocampus after brain strokes in humans [125] and rats [126].

Thus, the liaisons of excessive GCs /dysfunctional HPA axis with neuroinflammation, dangerous to the hippocampus, may be crucial for distant hippocampal damage in many brain diseases. Taking into account that the hippocampus controls both the cognitive functions and the emotional state, further research of potential links between GC signaling and the inflammatory processes in the brain and respective mechanisms is vital.

## Data Availability

Not applicable.

## Supplementary Materials

The following supporting information can be downloaded at: https://www.mdpi.com/article/10.3390/biomedicines10051139/s1, Table S1: Corticosterone changes in TBI models [127,128,129,130,131,132,133,134,135,136,137,138,139,140,141,142,143,144,145,146,147,148,149,150,151,152,153,154,155,156,157,158,159,160,161,162,163,164,165,166,167,168,169,170,171].

## Abbreviations

ACTH	adrenocorticotropic hormone
AMPA	α-amino-3-hydroxy-5-methyl-4-isoxazolepropionic acid
BDNF	brain-derived neurotrophic factor
CRH	corticotropin-releasing hormone
CS	corticosterone
DAMP	damage-associated molecular patterns
DG	dentate gyrus, hippocampal field
EPSP	excitatory postsynaptic potential
GABA	gamma-Aminobutyric acid
GCs	glucocorticoids
GR	glucocorticoid receptor
HPA	hypothalamo-pituitary axis
IL-1ß	interleukin 1 beta
IL-6	interleukin 6
iMR, iGR	intracellular cytoplasmic/nuclear receptors subtype
IPSC	inhibitory postsynaptic current
IPSP	inhibitory postsynaptic potential
LPS	lipopolysaccharide
MCAO	middle cerebral artery
mMR, mGR	membrane-associated receptors subtype
MR	mineralocorticoid receptor
NF-κB	nuclear factor kappa B
NMDA	N-methyl-D-aspartate
PTE	post-traumatic epilepsy
PV	parvalbumin
TBI	traumatic brain injury
TNFα	tumor necrosis factor alpha
VGCC	voltage-gated calcium channels
