# Supplementary material for: Brain Trauma, Glucocorticoids and Neuroinflammation: Dangerous Liaisons for the Hippocampus
Source: Biomedicines. 2022 May 15;10(5):1139. doi: 10.3390/biomedicines10051139 (PMC9138485; doi:10.3390/biomedicines10051139)
Supplement: Supplementary file 1 [file biomedicines-10-01139-s001.zip › biomedicines-1692357-supplementary.pdf]

Supplementary Table to the manuscript Ilia Komoltsev and Natalia Gulyaeva "Brain trauma, glucocorticoids and neuroinflammation: Dangerous liaisons for the hippocampus"

Table S1. Corticosterone changes in TBI models

	TBI model	Time after TBI	CS changes or manipulations affecting CS levels	Other findings	Ref.
1	Blast-induced TBI, rats	DPI2	Increased CS after blast acoustic cues, and more prominent in combination with TBI	Neuroinflammation and apoptosis in the ventral hippocampus. Anxiety behavior.	[129]
2	Blast-induced TBI, rats	2 months	Increased CS in stressed group and stressed+TBI group	An early increase in anxiety without memory impairment and increase of IL-6 in the hippocampus of stressed animals. In stressed+TBI group increased anxiety with spatial memory impairment, neuronal and glial cell loss, inflammation, hippocampal gliosis.	[128]
3	Blast-induced TBI, mice	DPI7–10	Increased restraint-induced CS in male and female mice	Attenuated restraint-induced CRF/c-Fos-immunoreactivity in PVN in males and enhanced in females after TBI. MR and GR gene expression not changed in the hippocampus.	[130]
4	CCI, rats	DPI1, 2, 3, 5, 7	on DPI 1 and 2. Decreased CS after treatment with dexamethasone.	Decreased expression of GR and MR on DPI2. Upregulation of MR expression induced by CS replacement.	[131]
5	CCI, rats	DPI3	Treatment with CS on DPI 1-3. Treatment with MR and GR antagonists 2 days before injury and on DPI1-3.	Low doses of exogenous CS after TBI increase MR activation, reduce apoptotic cells number and improve spatial memory. - High doses of CS overactive GR and induce pro-apoptotic pathways.	[132]
6	CCI, rats	DPI 1, 11, 19 and 29	Decreased CS.	Decreased testosterone and 11-deoxycorticosterone	[133]
7	CCI, rats	DPI3	Decreased CS after TBI.	Decreased MR expression and activation, spatial memory disturbance, apoptosis in the hippocampus. Fludrocortisone treatment improves TBI outcome.	[134]
8	CCI, rats	DPI7	Treatment with GCs during 7 days after TBI.	Increased cell loss in PVN after a high dose of methylprednisolone. Protective role of low-dose of corticosterone.	[135]
9	CCI, rats	4 h	Increased CS after TBI. Increased CS in ethanol-pretreated animals (dose-dependent)	Decreased IL-1β and TNFα in the brain of ethanol-pretreated animals.	[136]

10	CCI, rats	4 weeks	Blunted CS increase in response to restraint stress.	Febrile response to LPS, behavioral changes. Circadian body temperature during ethanol diet and withdrawal.	[137]
11	CCI, rats	24 h, DPI21	CS in night-tested rats compared to day group (TBI and sham).	Similar motor performance and spatial learning in day vs. night groups testing	[138]
12	CCI, rats	6 h, 24 h	Increased CS after TBI in males and pseudopregnant females.	Levels of steroids measured using gas chromatography in blood and brain.	[139]
13	CCI, rats	DPI28, 35	Blunted CS increase in response to restraint stress on DPI28 and facilitation by minocycline of CS response to restraint stress suppression in males and females. Decreased CS after TBI on DPI35 in males and females.	Prevention by minocycline of hyperthermia after TBI. Increased IL-1β in the female hippocampus.	[140]
14	CCI, rats	DPI1, 11, 19, 29	Decreased CS in TBI and sham operated rats. Lack of conjugated linoleic acids effect on CS levels.	Reversal of hypoadrenalism and limiting cognitive recovery after TBI induced by conjugated linoleic acids.	[141]
15	CCI, rats	1.5, 6, 12, 18, 24 h	Increased CS 1.5 and 6 h after TBI.	Increased ACTH 1.5 and 18 h after TBI. Increased hippocampal GR mRNA levels 24 h after sham operation by MR or GR antagonists. Decreased GR mRNA in the ipsilateral DG after TBI. Increased MR mRNA bilaterally in the CA3 of antagonist-pretreated animals.	[142]
16	CCI, rats	DPI 7, 21, 34, 54, 70	Attenuated stress-induced CS elevation on days 7, 21 after mild or moderate TBI. Persisted attenuation on day 70 after moderate TBI. Enhanced stress-induced CS elevation on days 34, 54, 70 after mild TBI.	Severity-dependent motor function loss (DPI12), cortical atrophy and hippocampal cell loss (DPI70). Severity-independent memory impairment (DPI15).	[143]
17	CCI, rats	DPI15-19, 28, 57–59	Increased CS in TBI animals. Increased CS in TBI and sham-operated animals treated with propylene glycol/ethanol.	Reversal of attenuating effects of moderate TBI induced by GR and GABA antagonists.	[144]
18	CCI, rats	DPI12	No change in plasma CS.	Alleviation of short-term memory impairments after treadmill exercise. Activation of pro-apoptotic pathway in hippocampal neurons.	[145]
19	CCI, rats	DPI42	Increased CS in ELS+TBI and ELS+sham group compared to control.	Increased IL-1β in the hippocampus in ELS+TBI group. Reversal of learning deficit in TBI group.	[146]

20	CCI, rats	DPI7, 35	Blunted CS increase in response to restraint stress. Suppression of CS responses to restraint stress by dexamethasone.	Suppression of ACTH responses to restraint stress by dexamethasone. In all rats, regardless of injury condition, CRH stimulated CS and ACTH. Ipsilateral neuronal loss in the hippocampal DG.	[147]
21	CCI, 7-day-old rats	3 weeks	Increased CS after TBI.	Negative correlation between serum CS and anxiety. Decrease in TBI-induced anxiety and serum CS, increased serum IGF-1 induced by progesterone treatment.	[148]
22	CCI, 7-days-old rats	DPI20	Increased CS after TBI.	Anxiety behavior in TBI rats. Decreased-↓ VEGF immunostaining score and neuronal density in prefrontal cortex	[149]
23	CCI, mice	3 months	Decreased CS after TBI (not in mice with depletion of factor XII).	Memory disturbances. GPIb-positive platelet aggregates.	[150]
24	CCI, mice and rats	1, 2, 4 weeks	Increased CS after TBI in mice after TBI or craniotomy.	Periorbital and paw allodynia in rats and mice.	[151]
25	Concussive head trauma, mice	DPI22	Increased CS and ACTH.	Increased anxiety-like behavior and HPA axis response to stress after CRF treatment. Decreased anxiety-like behavior and HPA axis response to stress after CRF antagonist treatment.	[152]
26	CSI, mice	DPI30	Increased CS in obese mice vs. lean controls. Decreased CS in obese mice after TBI vs. obese controls.	Anxiety behavior in obese mice. Decreased weight gain in obese TBI mice.	[153]
27	LFPI, rats	8 weeks	Increased CS in response to restraint stress. Prolonged CS elevation in ELS+TBI group.	Persistent learning and memory deficits. Cortical atrophy	[154]
28	LFPI, rats	DPI1, 3, 7, 14	Increased CS on DPI3 in blood, ipsilateral and contralateral hippocampus	Increased IL-1β, neuronal cell loss, CS-dependent neuroinflammation in the hippocampus	[56]
29	LFPI, rats	DPI5, 11-15	CS pre-treatment for 3 months before TBI (in drinking water) No differences in untreated aged rat plasma CS vs. treated adult rats before injury.	Chronic CS pre-treatment potentiated behavioral deficits after TBI.	[155]
30	LFPI, rats	4 h	No differences in CS levels between sham or TBI rats, with or without adrenalectomy+CS replacement. CS not detected in adrenalectomized rats without CS replacement.	Decreased NT-3 mRNA in the hippocampus of rats with LFPI or adrenalectomy.	[156]

31	LFPI, rats	DPI 3, 7, 14	Increased incidence of corticosteroid insufficiency in severe TBI group.	Decreased expression of endothelial tight junction protein. Increased BBB permeability. Apoptosis in the PVN.	[157]
32	LFPI, rats	4 h	7 days before TBI adrenalectomy with or without CS replacement (CS added in drinking water)	Increased BDNF mRNA in the hippocampus after TBI and/or adrenalectomy prevented by CS replacement.	[158]
33	LFPI, rats	DPI7, 14	Increased incidence of corticosteroid insufficiency in the group treated with methylprednisolone at a high dose on DPI7.	Apoptosis in the PVN, more prominent in methylprednisolone-treated rats. Decreased CS increase index in non-survivors after TBI.	[159]
34	LFPI, rats	DPI1, 3, 7, 14	Increased CS on day 3 in the ipsilateral and contralateral hippocampus	Cytokine expression in ipsilateral and contralateral hippocampus. Microglial activation and neurodegeneration in the hippocampus of both hemispheres. No significant changes in GR (Nr3c1), MR (Nr3c2), Crh and its receptors (Crhr1 Crhr2) mRNA expression.	[55]
35	LFPI, rats	DPI28-32, 35-39	Similar CS levels in TBI and sham-operated groups before exercises. Increased CS and ACTH after forced exercises.	Increased BDNF after voluntary exercises.	[160]
36	LFPI, rats	DPI0-4, 7-11	Increased CS and ACTH after forced exercises.	Decreased GR in the hippocampus after forced exercises.	[161]
37	LFPI, mice	DPI3	Decreased CS in TBI mice.	Increased corticosteroid insufficiency after high-dose dexamethasone treatment.	[162]
38	LFPI, mice	DPI3	Decreased CS in TBI+sleep deprivation vs. sham-operated group.	Neuroinflammation in neocortex. Anxiety-like behavior.	[163]
39	mFPI, rats	6, 24 h, DPI7, 14, 28, 54	Increased CS on DPI1. Decreased CS on DPI7, 14, 28, 54 vs. baseline. Blunted CS response to restraint stress on DPI28, 54 vs. DPI7.		[164]
40	mFPI, rats	2 months	Decreased CS. Blunted CS increase in response to restraint stress.	No changes in testosterone level. Altered complexity of neuronal processes in the PVN.	[165]
41	mFPI, rats	DPI28, 29	Increased CS after deprivation compared to stimulation therapy.<br>Increased CS in TBI group on DPI29.		[166]

42	mFPI, rats	4 weeks	Increased CS by whisker nuisance task after TBI vs. sham-operated group.	Chronic injury-induced sensory sensitivity on whisker nuisance.	[167]
43	mFPI, rats	DPI1-2	No effect of sleep disruption on CS levels in TBI group.	No effect of sleep disruption after TBI on motor and cognitive performance	[168]
44	Polytrauma (blast overpressure + hemorrhage), rats	0, 3, 72 h	Increased CS and ACTH 3 and 72 h after polytrauma.	No effect of pre-stress on CS levels after polytrauma.	[169]
45	Stab wounding of a hemisphere, rats	DPI23	No effect of exercise on CS levels.	Worsening motor impairment by early exercise.	[170]
46	WD, rats	6, 48 h, DPI14	No differences between effects of 100 and 120 cm WD.	Transcriptomic analysis performed in hippocampal and cerebellar tissue.	[171]
47	WD, rats	DPI27	No differences between CS levels in TBI and sham-operated groups, treated or untreated with Etazolate	Decreased cAMP, pCREB, BDNF. Depression-like behavior. Etazolate attenuated behavioral deficits and biochemical changes.	[172]
48	WD, mice	2, 4 weeks	Increased CS 2 weeks after TBI. Aggravation of CS elevation by social isolation.	Memory impairment in isolated and social mice after TBI. Aggravation of excitotoxic damage to the hippocampus by isolation.	[173]

Abbreviations
ACTH - adrenocorticotropic hormone; BBB - blood brain barrier; BDNF - brain-derived neurotrophic factor; cAMP - 3′,5′-cyclic adenosine monophosphate; CCI - controlled cortical impact model; CRF - corticotropin-releasing-factor; CRH - corticotropin-releasing hormone; CS - corticosterone; CSI - controlled skull impact; DG - dentate gyrus; DPI - days post-injury; ELS - early life stress; GPIb - Glycoprotein Ib; GR - glucocorticoid receptor; IGF-1 - insulin-like growth factor 1; IL-1ß - interleukin 1 beta; LFPI - lateral fluid percussion model; mFPI - midline fluid percussion model; MR - mineralocorticoid receptor; NT-3 - neurotrophin-3; pCREB - cAMP-triggered phosphorylation of cAMP response element binding protein; PVN - paraventricular nucleus; TBI - traumatic brain injury; TNFα - tumor necrosis factor alpha; WD - weight drop model.
